# The metamorphosis of the developing cerebellar microcircuit

**DOI:** 10.1016/j.conb.2011.01.009

**Published:** 2011-04

**Authors:** Ingrid van Welie, Ikuko T Smith, Alanna J Watt

**Affiliations:** Wolfson Institute for Biomedical Research, University College London, London, WC1E 6BT, United Kingdom

## Abstract

The cerebellar cortical circuit with its organized and repetitive structure provides an excellent model system for studying how brain circuits are formed during development. The emergence of the mature brain requires that appropriate synaptic connections are formed and refined, which in the rodent cerebellum occurs primarily during the first three postnatal weeks. Developing circuits typically differ substantially from their mature counterparts, which suggests that development may not simply involve synaptic refinement, but rather involves restructuring of key synaptic components and network connections, in a manner reminiscent of metamorphosis. Here, we discuss recent evidence that, taken together, suggests that transient features of developing cerebellar synapses may act to coordinate network activity, and thereby shape the development of the cerebellar microcircuit.

## Research highlights

► The developing cerebellar circuit exhibits transient synaptic elements. ► Network activity and synaptic plasticity are shaped by such transient circuit elements. ► Transient circuit features may be pivotal in the development of cerebellar circuits. ► The cerebellum is a highly attractive model system for the study of circuit development.

## Introduction

In present day neuroscience, much effort is devoted to mapping the connections within the circuits of the mature brain — the connectome (e.g. [1]) — in the hope to shed light on how the brain works. Another approach to understanding brain function is to study how the brain develops, since uncovering how something is built can illuminate how it functions. Additionally, gaining an understanding of how brain circuits develop helps unravel those instances when neuronal development goes awry, such as during developmental disorders and diseases. For example, many congenital ataxias are characterized by developmental malformations in the cerebellum. Thus, a better understanding of healthy cerebellar development may lead to new therapeutic strategies to treat these devastating disorders [2].

**Figure 1:**
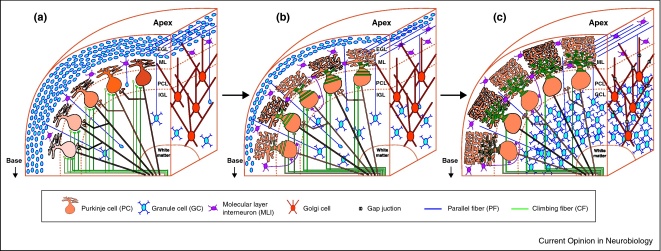
Metamorphosis of the cerebellar cortical circuit. The developing cerebellar circuit undergoes dramatic changes during postnatal development. Major components of the cerebellar circuit are illustrated at different developmental stages, corresponding to **(a)** postnatal week 1, **(b)** postnatal week 2, and **(c)** adult in rodent. A similar developmental sequence has been observed for some circuit elements in other species, but the time course differs. PCs (orange) exhibit traveling waves in the first postnatal week (illustrated by orange colour gradient in a), make synapses onto other PCs in early development (a), which are reduced in number by postnatal week 2 (b), and absent in adult. PCs receive multiple somatic CF inputs (green) in the first postnatal week, with one winner CF innervating the dendrites by the second postnatal week, with at least 1 weaker somatic inputs remaining (b). Monoinnervation of PCs by CFs is seen in adult (c). Granule cells (blue) migrate from the EGL, to the IGL during the first (a) and second (b) postnatal week. MLIs (purple) innervate PCs and each other in the second postnatal week (b), and in the adult (c). Golgi cells (red) exhibit gap-junction coupling in the adult (c), although possibly earlier as well (not shown).

One of the defining features of the cerebellar cortex is its organized and repetitive laminar organization that is highly conserved across species. This brain region is composed of a mere handful of cell types that are relatively easily identified: Purkinje cells (PCs), granule cells (GCs), Golgi cells, Lugaro cells, unipolar brush cells and basket and stellate cells, with the last two often grouped together as molecular layer interneurons (MLIs). In the adult, these cell types form stereotypical connections, organized into a simple three-layered laminar structure, consisting of the molecular layer, the PC layer, and the GC layer (Figure 1). Thus, from the point of view of circuit formation, we have a relatively good understanding of the end point in cerebellar development, making the cerebellum a good model system for circuit development. Insights gained in cerebellar development may help us understand circuit development in other brain regions as well.

Brain development involves at least two distinct processes: (1) neurogenesis and migration of neurons and/or their precursors, and (2) the formation of synapses and the emergence of neuronal circuits. Much work has shed light on the former process of cell neurogenesis and migration in the cerebellum (for review see [3,4]). In brief, most cell types have differentiated and migrated roughly to their mature location by the first postnatal week, except for GCs which undergo a long period of neurogenesis in a transient cell layer, the external GC layer that extends into the third postnatal week in rodents [3] (Figure 1).

**Figure 2:**
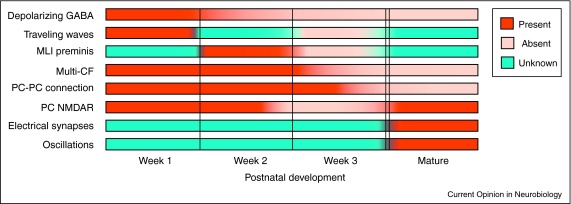
Postnatal timeline of circuit metamorphosis. Multiple changes occur in the developing cerebellar circuit involving both up-regulation and down-regulation of synaptic elements and the formation and elimination of connections. The timelines of the circuit alterations discussed in this review are shown here, with developmental time on the *X*-axis. Each vertical bar represents ∼1 postnatal week in rodents, separated by a double line from the adult profile. Red = present, cream = absent, and blue = unknown.

This review focuses on the latter developmental process, the emergence of neuronal circuits through the formation and refinement of synaptic connections. Recent work suggests that rather than being a roughly drawn version of the mature cerebellar circuit, the scale of circuit reorganization in the developing cerebellum is akin to a process like metamorphosis. Like a frog egg hatches into a tadpole before ultimately becoming a frog, the synaptic connections underlying the cerebellar circuit pass through a variety of developmental stages that include the appearance and disappearance of ontogenetically transient synaptic components and connections, before maturing into the adult circuit (Figures 1 and 2). We suggest that these transient features may set circuit activity, regulate synaptic plasticity, and alter network connectivity, and that these transient features may be pivotal in the developing circuit to guide the emergence of the mature cerebellum.

### E_GABA_ in the developing cerebellum

GABA is the main inhibitory neurotransmitter and elicits hyperpolarizing postsynaptic responses in the mature brain. In the developing brain, however, responses to GABA are often depolarizing [5]. In the juvenile cerebellum, depolarizing GABA has been observed in those cells in which it has been measured, including PCs [6^••^,7], GCs [8,9^•^], and MLIs [10] (Figure 2). The GABA reversal potential (E_GABA_) is thought to arise largely due to a differential expression of Cl^−^ transporters, with the Na^+^–K^+^–Cl^−^ transporter NKCC1 expression dominating in the young central nervous system (CNS) and expression of the K^+^–Cl^−^ transporter KCC2 dominating in the mature CNS [5]. Depolarizing GABA is required for normal brain development, as it contributes to the morphological maturation of neurons [11], and neuronal circuits [12,13]. Depolarizing GABA can drive juvenile neurons to fire action potentials [5] and conversely, neuronal activity can regulate E_GABA_, by either specific patterns of synaptic activation [14,15^•^], or alterations in postsynaptic activity levels [11] via changes in intracellular Ca^2+^ [16]. How activity-dependent changes in Ca^2+^ are translated to changes in the surface expression of Cl^−^ transporters is not known, although a study in cerebellar GCs suggests that it may involve microRNAs [9^•^].

Surprisingly, two recent studies have suggested that the depolarizing GABA widely observed in the young brain is non-physiological, and that GABA is in fact hyperpolarizing in juvenile animals [17^••^,18]. The crux of this argument is that the typical experimental artificial cerebrospinal fluid (aCSF) used in *in vitro* experiments is based on adult brain CSF, which may not mimic the CSF of the young mouse brain well. Neonatal brains contain elevated levels of several metabolic substrates, in particular the ketone body β-hydroxybutyrate (BHB) compared to adult brains [19]. These two studies tested what effect elevated BHB in juvenile brain might play on E_GABA_ during development. They found that BHB caused E_GABA_ to hyperpolarize when applied to young hippocampal [17^••^] or cortical neurons [17^••^,18] — at an age when E_GABA_ was otherwise depolarizing. Hyperpolarizing effects on E_GABA_ [18] or a reduction on network activity [20] were also attributed to other metabolic substrates including lactate and pyruvate. Some, however, have questioned the physiological significance of these findings [21^••^,22]. The authors concluded that physiological E_GABA_ may be hyperpolarizing throughout development due to the presence of BHB in young brain tissue.

In response to these controversial findings, two even more recent studies have also looked at the effect of BHB on E_GABA_ in early development [21^••^,23], and have drawn different conclusions. In their hands, BHB does not hyperpolarize E_GABA_ in young hippocampal [21^••^], or cortical neurons [21^••^,23], nor does it alter the spontaneous activity exhibited in these young circuits [21^••^]. Several technical explanations for the discrepancies between these studies have been raised [21^••^], and remain to be further investigated. On a cautionary note, multiple studies have reported the presence of the bioactive contaminant dibenzylamine in commercially purchased BHB (e.g. [24–26]). In one recent study [21^••^], the authors found that BHB that is contaminated by dibenzylamine causes E_GABA_ to hyperpolarize. The presence or absence of contamination in BHB was not determined in other recent studies (e.g. [17^••^,18,23]). In our opinion, the current evidence favors the view that GABA is depolarizing in neonates. Regardless, this controversy is instructive, as it reminds us that the juvenile brain differs from the mature brain in many ways, including its metabolism.

### Transient presynaptic miniature currents

While changes in E_GABA_ may affect multiple synapses, other developmentally transient synaptic features are thought to be synapse-specific. One such mechanism, transient presynaptic miniature currents (preminis), has recently been described in developing MLIs of the cerebellum. Preminis are miniature currents that arise due to the activation of GABA_A_ receptors in presynaptic terminals in the axons of MLI neurons, and exist in the second postnatal week (and possibly earlier), but are absent by the third postnatal week [27^••^] (Figure 2). Intriguingly, the frequency of preminis increases with subthreshold presynaptic membrane depolarization, a phenomenon mediated by voltage-dependent Ca^2+^ entry, which suggests that premini frequency directly reflects the activity of the presynaptic cell and thus is part of a positive feedback loop regulating neurotransmitter release [27^••^].

Although the function of preminis is at present unknown, some intriguing possibilities exist. Because GABA is depolarizing at the ages preminis are observed (although see above, and [10,27^••^]), preminis may enhance depolarization at boutons and augment transmitter release. Such a positive-feedback loop might help define the functional axonal segments that are retained during axonal refinement, while those that do not exhibit preminis are pruned. Another idea — not mutually exclusive — is that preminis contribute to excitation at the soma, possibly leading to changes in gene expression, protein synthesis, or axonal transport of specific proteins [28]. It is noteworthy that preminis are easy to miss experimentally, as they are very small when measured at the soma. This hints that preminis may be expressed more widely in the developing brain, but have hitherto been overlooked.

### Expression of NMDA receptors in Purkinje cells

At glutamatergic synapses, NMDA receptors (NMDARs) are thought to be crucial for several forms of plasticity, including some forms of long-term potentiation (LTP), depression (LTD), and spike-timing-dependent plasticity (STDP) [29]. A common pattern of development in many brain regions is that, in addition to the obligatory NR1 subunits, NMDARs containing NR2B subunits predominate early in development, while NR2A subunit-containing NMDARs predominate later in development. Early NR2B-containing NMDAR exhibit enhanced Ca^2+^ influx, and are thought to contribute to activity-dependent remodelling and the development of cortical circuitry [30]. Typical switches from NR2B to NR2A containing NMDARs in developing GCs have been well characterized, although as GCs mature further, they incorporate NR2C subunits in their NMDAR, reducing the Mg^2+^ sensitivity of the receptors [31]. Until recently, NMDARs were believed to be largely absent in mature PCs, with expression limited to the early postnatal development [32], and then declining during the second postnatal week.

Surprisingly, recent studies show that after the initial decline in NMDAR expression from the first to second postnatal week in PCs, NMDAR currents then increase from the third postnatal week onwards in PCs, mediated by expression of both NR2A and NR2B subunits [33^•^,34^•^] (Figure 2). A functional role for both presynaptic [35] and postsynaptic NMDARs [36] has been shown at climbing fiber (CF)–PC synapses. Postsynaptic NMDARs are specifically involved with the induction of LTD (induced by combined parallel fiber (PF) and CF activation) but not LTP (induced by PF stimulation alone) at PF–PC synapses [36].

What role might the transient elimination of NMDARs serve in the development of PCs? Why does the developmental profile of NMDAR expression differ in PCs from its typical pattern in the developing brain? Since the decline of NMDAR expression appears to correlate inversely with the period when the majority of PF–PC synapses are formed (see Box 1), perhaps the absence of NMDARs is important for the establishment of PF–PC innervation. Another explanation would be that the behaviourally relevant function mediated by NMDAR-dependent LTD is not established until the maturation of the circuit. It will be interesting to learn both how this unique ontogenetic regulation of NMDARs takes place and what it serves functionally.

### Climbing fiber — Purkinje cell synapse refinement

One of the classic examples of circuit refinement in the CNS occurs in the cerebellum: the pruning of CF inputs onto PCs. In rodents, each PC is initially multiply innervated by several CFs. These supernumerary CFs go through a process of competitive elimination until only a single fiber remains by the end of the third postnatal week [37] (Figure 2). The gradual elimination of all-but-one CF is preceded by a subtle segregation of synaptic strength among multiple inputs, where the eventual winner gains strength [38]. The winning fiber then continues to grow in strength as it translocates from the perisomatic area of the PC to its proximal dendrites [39^••^], while other smaller inputs are progressively eliminated (Figure 1).

The selection and maturation of the winner CF, and the elimination of the smaller inputs, is influenced by neural activity. In acute brain slices, paired activation of a postsynaptic PC and its winner CF induces LTP of the synapse. LTP exclusively occurs at the ‘winner’ synapse while a similar protocol induces small inputs to undergo LTD [40^••^]. The observed LTP is mediated by postsynaptic Ca^2+^ signaling [40^••^] and may facilitate the competition as well as further translocation of the ‘winner’ CF to the dendrites.

Presynaptic activity is not the only factor in the CF–PC circuit refinement: the postsynaptic PC also plays a role in the process. Overexpression of a chloride channel in PCs perturbs their excitability and results in persistent multiple innervation by CFs [41^•^]. It should be noted, however, that changes in the postsynaptic PC activity could ultimately influence the activity level of the inferior olive via the PC — deep cerebellar nuclei — inferior olive loop [42]. Since CFs are axons of olivary neurons, manipulating postsynaptic PC activity might thus alter presynaptic CF activity as well. Another key factor in CF elimination is PF synapses, the other main excitatory input to PCs (Box 1 reviews recent studies elucidating the mechanism of PF–PC synaptogenesis). Although the PC dendritic territories occupied by PFs and CFs are clearly delineated, with the former innervating the distal dendrites and the latter the more proximal regions, these territories are actively maintained through heterosynaptic competition. Regression of one territory swiftly results in the expansion of the other, even in adulthood [43,44,45^•^]. Additionally, recent evidence indicates that GABAergic synapses may play a role in early CF competition, when E_GABA_ is depolarizing (see above, and [46]), illustrating the complicated interplay between multiple synaptic pathways in the developing cerebellum.

### Transient Purkinje–Purkinje cell synapses

**Figure 3:**
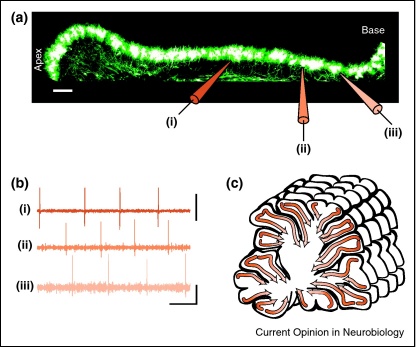
Traveling waves mediated by transient Purkinje–Purkinje cell synapses. During the first postnatal week, PC activity is coordinated to produce traveling waves moving from the tip of the lobule towards its base (illustrated by orange gradient). **(a)** Image from young animal (P4) showing location of recording electrodes. Scale bar: 50 μm. **(b)** Traces showing wave-like activity across PCs recorded from neurons indicated in (a). Scale bars: 1 nA (top trace), 100 pA (bottom two traces) and 100 ms. **(c)** Schematic illustration showing how individual waves travel down each lobule from apex to base. Adapted from Ref. [6^••^].

Another striking example of ontogenetically transient connections is the transient synaptic connection made by PCs onto other PCs (Figures 1 and 2). These monosynaptic GABAergic connections are prevalent during the first postnatal week, exhibit reduced connectivity in the second postnatal week, and are nearly completely pruned by the third postnatal week of development [6^••^,47]. PC axon collaterals mediating these connections project asymmetrically within the sagittal plane, towards PCs lying away from the apex of the lobule in which the cell lies, forming chains of connected PCs. These chains form a substrate for traveling waves in the juvenile cerebellum [6^••^] (Figure 3), which are observed during the first postnatal week when E_GABA_ is depolarizing [6^••^,7] (Figure 2). It is worth noting that, if E_GABA_ were hyperpolarizing in young PCs (see above, and [17^••^,18]), waves of activity would still be observed except traveling in the opposite direction [6^••^].

Patterned network activity mediated by GABAergic transmission is a feature of many developing neuronal circuits [8], including the retina, the spinal cord, the cochlea and the hippocampus, and is thought to take part in the developmental refinement of circuits [48]. The presence of travelling waves in the developing cerebellum, and their similarities to early network activity observed in these other brain regions, suggest that these cerebellar waves may be important in ensuring proper development of the cerebellar circuit.

What function might these early waves serve in the developing cerebellum? Traveling waves produce structured firing between neighbouring PCs that resembles the ‘pre-before-post’ pattern of activity required to induce some forms of long-term synaptic plasticity, such as STDP. Other GABAergic synapses exhibit STDP [14], where it plays a direct role in regulating synaptic properties like E_GABA_ (see above, and [14,15^•^]). The capacity for activity-dependent plasticity at PC–PC synapses is yet unexplored, but could be a mechanism important to shape the cerebellar network.

Additionally, the structured firing between neighbouring PCs due to travelling waves produces oscillatory activity at theta frequency (4–9 Hz). Although mechanistically distinct, this early activity might be functionally related to the theta oscillations observed in adult cerebellum [49,50]. In addition to producing oscillations, however, a travelling wave also produces temporally ordered spiking that has a sense of direction, moving across the lobule from its tip to its base (Figure 3c). Thus, traveling waves may enable timing or positional computations in the developing cerebellar circuit.

As we have described earlier, traveling waves are observed at a time when the immature cerebellar circuit is undergoing radical changes, morphing into the mature circuit and travelling waves might play a role in this process. Looking at the output of the cerebellar cortical circuit, the synapses made by PC axons onto neurons in the deep cerebellar nuclei are established at the end of the first postnatal week [51], at the tail end of the period of travelling waves. However, PC axons are present in the deep cerebellar nuclei several days before they make functional synapses when travelling waves may be most prevalent. This suggests that PC axons may undergo refinement in the deep cerebellar nuclei after initial innervation but before functional synapses are made [51], and that this process might involve these travelling waves [6^••^].

### Electrical synapses

Developing circuits in the brain use not only chemical synapses to communicate, but electrical synapses as well. In the embryonic retina, for example, communication via gap junctions mediates circuit formation by contributing to proper cell type distribution [52], cell proliferation [53], and retinal wave propagation [54]. Electrical synapses transmit presynaptic voltage signals and small molecules rapidly and therefore may mediate network synchronization. Due to the often cell-specific expression of gap junctions, activity may be coordinated in sub-networks of particular cell types, within the larger developing neuronal circuit. Although there is little evidence for functional electrical synapses in the developing cerebellum to date, dye-coupling between PCs, which suggests the presence of gap junctions, has been observed in juvenile cerebellar organotypic slices [55].

Additionally, several gene expression studies suggest that electrical synapses may be enriched in the developing cerebellar circuit. Connexins are the main genes encoding gap junction proteins in vertebrates, and two widely expressed forms are seen in the developing cerebellum: connexins 36 (Cx36) and 45 (Cx45). Cx36 mRNA expression is observed in the first week of postnatal life in the molecular and inner GC layer [56]. Cx36 expression is developmentally regulated, gradually declining from birth to the end of the third postnatal week [57]. Cx45 is also expressed in several cell types in the cerebellum in this same developmental window, but by the end of the third postnatal week is only expressed in MLIs [57].

Given the combination of abundant and transient expression in many developing cells of the cerebellar cortex, one may suspect that connexins play a key role in the development of the cerebellar circuit. Surprisingly, however, functional gap junction coupling has only been shown to date in young adult tissue in MLIs and in Golgi cells [58,59^••^,60^••^] (Figure 2).

Electrical coupling is generally thought to contribute to oscillations in the brain. Consistent with this idea, coupling between MLIs results in roughly synchronous firing across neurons, independent of synaptic inputs, which can be amplified by intrinsic conductances [58]. Electrical coupling between Golgi cells is mediated by Cx36 and may result in low-frequency oscillatory activity and resonance in Golgi cell networks [59^••^,60^••^]. In the mature cerebellum, oscillations occur at multiple frequencies including theta (4–9 Hz [49,50]) and beta (10–30 Hz [61,62]) in the GC layer, and gamma (30–80 Hz [63]) in the PC layer (Figure 2). Additionally, even higher frequency oscillations have been reported in the PC layer (80–160 Hz [63]; 160–260 Hz [64]). It will be interesting to determine whether the observed enriched connexin expression during development is correlated with early oscillatory activity, arising from functional electrical synapses.

## Conclusions and future directions

In this review, we have examined several transient features of connectivity in the developing cerebellar microcircuit (summarized in Figures 1 and 2). We propose that some of the processes we described likely serve to enable proper wiring of the major connections in the adult circuit. For example, changes in E_GABA_, as well as the transient expression of presynaptic GABA_A_R may set activity levels within appropriate ranges for subsequent developmental changes, like structural refinements, to occur. Changes in synaptic composition, such as the transient expression of presynaptic GABA_A_Rs and absence of postsynaptic NMDARs during the second postnatal week will likely affect synaptic plasticity at those synapses, which may be instrumental during synaptic refinement. Although we have focused on the developing cerebellum, some ontogenetic events, like depolarizing GABA [5], are ubiquitous across the juvenile brain; indeed, there is good evidence to suggest that other developing brain regions undergo analogous processes of circuit metamorphosis [12,48]. We argue that with its repetitive and well-organized mature circuit, the cerebellum makes an exquisite model for studying how transient developmental features lead to proper formation of the mature circuit.

Some of the developmental processes we described, including CF–PC pruning and PC–PC connections involve transient restructuring of the developing circuit, and may affect network activity. Circuit rewiring is energetically costly, which argues that transient connections may be pivotal in development. In support of this argument, it appears that passing through at least some of these developmentally transient stages is critical for mature cerebellar function. For example, persistent multiple CF innervation into adulthood is associated with impaired motor control [42,46]. Many developing sensory as well as motor-related brain regions exhibit spontaneous transient activity, which may be important for the rough formation of developing circuits before they are further refined by sensory input [12,48]. Devising new paradigms to study CF–PC pruning and early traveling waves *in vivo* and in a behaviorally relevant context may allow us to gain further insight into the role of these transient connections and the role they serve in cerebellar development.

The recent emergence of different types of genetic and optogenetic tools may allow more detailed future studies of these transient phenomena in cerebellar development. As more cell type-specific promoters are identified for neurons in the cerebellum, application of these tools will help elucidate the roles of different transient connections in cerebellar circuit development, and may help us understand if these transient developmental features are interrelated or work synergistically.

In conclusion, we suggest that the cerebellar circuit does not simply develop from a rough outline to a filled-in version of the adult cerebellum, but rather undergoes a series of developmentally regulated steps involving transient connections and synaptic components that may work together to guide the emergence of — or metamorphosis to — the adult cerebellar circuit (Figure 1).

## References and recommended reading

Papers of particular interest published within the period of review have been highlighted as:

• of special interest
•• of outstanding interest

## Box 1 Parallel fiber—Purkinje cell synaptogenesis: the molecular building blocks of a synapse.

Exciting new work is unraveling the molecular underpinnings of PF–PC synapse development. The glutamate receptor δ2 subunit (GluD2), is unique among glutamate receptors as it has no binding affinity to glutamate, nor does it exhibit any functional currents [65]. The selective expression of GluD2 in PCs, and its involvement in ataxia [66], has inspired a series of studies aimed to investigate its role in cerebellar function. Within PCs, GluD2 localizes to the distal dendritic spines innervated by PFs [67]. It is required for PF–PC synapse formation, since genetic deletion of GluD2 results in a severe reduction in the number of PF–PC synapses and abnormal extension of CF–PC synaptic territory out into the distal spines abandoned by PFs [68,69].

Although long regarded as an orphan receptor, recently, a glycoprotein secreted from granule cells called precerebellin 1 (Cbln1) was found to be the binding partner of GluD2 [70^••^]. Subsequently, the presynaptic binding partner of Cbln1 has been recently identified as the synaptic cell-adhesion protein Neurexin [71^•^]. Thus, presynaptic-derived Cbln1 tethered to the terminal via Neurexin, together with postsynaptic GluD2 works as a bidirectional synapse organizer. This binding of Cbln1 to GluD2 triggers synapse formation and recruits several postsynaptic density proteins [70^••^]. Interestingly, the secretion of Cbln1 is regulated in an activity-dependent manner. An overall increase in neural activity results in decreased expression of Cbln1, and a reduction in the number of excitatory synapses on PC dendrites [72]. The activity-dependent regulation of Cbln1 release may function homeostatically to stabilize activity levels in the developing cerebellum.
